# Supplementary material for: Patterns of item nonresponse behaviour to survey questionnaires are systematic and associated with genetic loci
Source: Nat Hum Behav. 2023 Jun 29;7(8):1371–87. doi: 10.1038/s41562-023-01632-7 (PMC10444625; doi:10.1038/s41562-023-01632-7)
Supplement: Supplementary file 1 — Heckman Supplementary Note, Frequently Asked Questions and Figs. 1–10. [file 41562_2023_1632_MOESM1_ESM.pdf]

nature human behaviour

Article https://doi.org/10.1038/s41562-023-01632-7

Patterns of item nonresponse behaviour to survey questionnaires are systematic and associated with genetic loci

In the format provided by the authors and unedited

This PDF file includes:

Heckman Supplementary Methods

Supplementary Discussion: Frequently Asked Questions

Supplementary Figures 1 to 10

Supplementary References

Correspondence to: rwedow@purdue.edu and andrea.ganna@helsinki.fi

SUPPLEMENTARY METHODS

Heckman correction of phenotypic associations

As additional proof of concept for Heckman correction with the nonresponse (NR) factors we evaluate correction of phenotypic associations. Specifically, we correct association of fluid intelligence (FI) with use of paracetamol (UKB fieldID 20003, response code 2038460150), height (UKB fieldID 50), and leg fat percentage (UKB fieldID 23111) at baseline assessment. We choose these three variables since they did not themselves allow item nonresponse and each shows significant phenotypic association with FI that we might expect to be at least partially artifactual. For these phenotypic associations Heckman correction was implemented in the Python "statsmodels" package, with the sign of Heckman's λ reversed to remain consistent with the normal specification of Heckman correction.

The predicted selection likelihood for FI based on NR factors, missingness-related variables, and covariates (Methods) is strongly associated with FI in the models with paracetamol (Heckman λ coefficient=2.874[0.042]), height (λ coefficient=2.810[0.042]) and leg fat (λ coefficient=2.913[0.043]). In all three cases Heckman correction leads to substantially reduced estimates of the association with FI (uncorrected regression beta=-0.228[0.019], corrected regression beta=-0.069[0.0183] for paracetamol; uncorrected beta=0.030[0.001], corrected beta=0.018[0.001] for height; uncorrected beta=-0.022[0.001], corrected beta=0.004[0.001] for leg fat). This is consistent with our hypothesis that at least part of the observed phenotypic association of FI with these variables is attributable to bias from nonrandom missing data.

Heckman correction of GWAS with only nonresponse factors

To evaluate how sensitive the Heckman-corrected GWAS results for FI are to the choice of selection model, we also perform GWAS of FI using a selection model that includes only the nonresponse factors and covariates (i.e. omitting health and SES-related variables).

The first stage selection model shows somewhat weaker power to explain the missingness after omission of the health and SES variables (pseudo-r2=0.027). The resulting missingness prediction is then more weakly associated with FI (Heckman λ coefficient=1.420[0.052], $p=2.08\times10^{-164}$). Heckman corrected GWAS of FI with this reduced selection model (N=92532) shows smaller differences from uncorrected GWAS of the same samples. Genetic correlation between the corrected and uncorrected GWAS

minimally differs from 1 (r_g=0.9991[9.22x10^{-5}], p=8.17x10^{-23} for test of r_g=1). Among significant loci, 11 of the 14 lead SNPs from the uncorrected GWAS remain genome-wide significant in the Heckman-corrected GWAS, with limited attenuation in estimated effect sizes (Deming regression slope = 0.987[0.005], p=0.0139 for test of slope=1; **Suppl. Tab. 14**). Genetic correlations of FI with PNA and IDK are also modestly reduced in this Heckman-corrected GWAS (uncorrected GWAS r_g=-0.40[0.04], corrected r_g=-0.37[0.04] for PNA; uncorrected GWAS r_g=-0.27[0.03], corrected r_g=-0.24[0.03]).

Overall, the impact of Heckman correction on GWAS of FI is much smaller when the selection model is restricted to only NR factors and covariates. On the other hand, the impact of the Heckman correction (e.g., smaller assocaitons with of top hits, reduced r_g with PNA and IDK) largely follows similar trends, just with weaker magnitude. This may indicate that correction of GWAS with only the current NR factors is underpowered, consistent with the weaker pseudo-R^2 of the selection model, and thus stronger modeling of nonresponse behavior is required to fully address bias from nonresponse in GWAS.

Interpretation of Heckman correction results

Although it is tempting to adopt the results of GWAS with Heckman-correction as definitive new GWAS estimates, that would be premature for the current preliminary results for FI. We anticipate that there are a number of caveats to the interpretation of GWAS with Heckman correction that will need to be addressed before fully embracing Heckman-corrected GWAS results as the primary GWAS for any given phenotype. Instead, we evaluate the current results as a proof of concept for the potential impact of missingness corrections on GWAS, with a focus on qualitative trends rather than specific quantitative results. We elaborate here on the caveats that will need more careful evaluation before being adopted in applied analyses.

First and foremost, Heckman correction provides no guarantees that the resulting regression estimates will be unbiased. The two step Heckman estimator only corrects for missingness that can be explained by observed variables. In other words it addresses the possibility of data being missing at random (MAR[1]; e.g., $\gamma \neq 0$ in **Fig. 1**) but not missing not at random (MNAR; $\theta \neq 0$ in **Fig. 1).** By extension, the effectiveness of the correction depends on power in the selection model, and thus will be affected by sample size, error in predictor variables, and availability of data for desired auxiliary variables, among other features.

Relatedly, the components of missingness-related bias that will be corrected by the Heckman estimator (in full or in part) will depend on the content of the selection model. This implies that there may be some risk of collider bias[2] or other confounds being introduced by misspecification of the selection model. Given that missingness-related bias can be thought of as a form of collider bias[3], future work will likely need to focus on best practices for constructing the selection model in the context of GWAS in order for Heckman correction to be a viable solution for future GWAS.

Given these uncertainties, we focus on Heckman correction results as an indicator for the possible presence of missingness-related bias and initial evidence for the direction of that bias. The test of the coefficient for Heckman's λ in the response regression provides a direct test of whether the modeled selection is informative to the phenotype. If Heckman's λ suggests the presence of nonrandom missingness then there's at least the potential for bias in the results of uncorrected GWAS (or other regression). In that case, comparison of the corrected and uncorrected results can provide at least qualitative information on the possible direction of the bias, even if the resulting corrected estimate isn't fully unbiased. Evaluating the sensitivity of the implied direction of bias to the choice of Heckman selection model variables may also help with evaluating concerns about biases induced by the specification of the selection model as described above.

Finally, we note that Heckman correction isn't the only possible method for correcting bias from nonrandom missingness. Alternative approaches using sampling probabilities[4], full maximum likelihood models for the missingness mechanism[5], or other instrumental variable-based adjustments[6] all have potential for addressing nonrandom missingness. Evaluation of these methods in the context of GWAS will need to balance their effectiveness for bias correction with their computation complexity, data requirements, and ease of model specification, including for potentially complex missingness mechanisms. We're hopeful that future work will provide stronger recommendations about how to best address bias from missing data in GWAS.

SUPPLEMENTARY DISCUSSION

Frequently Asked Questions (FAQ)

Background/Motivation

1. **This study focuses on "item nonresponse". What is "item nonresponse"?**

When no substantive answer is provided by a study participant in a questionnaire, such as an "I don't know" response, researchers call this 'item nonresponse'. In relation to its observability as a behavior, item nonresponse highlights the complex interaction between analyzing survey designs for questionnaires and a respondent's cognitive processes. Item nonresponse can be thought of as an intermediate on a scale between providing complete information and complete nonparticipation. In this study, item nonresponse was recorded through a participant's propensity to respond with "Prefer not to answer" (PNA) or "I don't know" (IDK) to survey questions distributed by the UK Biobank.

2. **What are the goals of this study? | Why did you do this study?**

The goal of this study was to use genetic information to better understand item nonresponse behavior. Item nonresponse has relevant implications related to both behavioral choices made by study participants and to statistical concerns over missing data in research. Nonresponse behavior has also been correlated with some heritable traits such as health status and educational attainment in addition to other psychological and personality traits such as low self-confidence. Moreover, these individual differences in item nonresponse rates could be related to the questionnaire content itself or that characteristics of the study population.

Similar participant patterns are observed for study participation and item nonresponse. For example, participants with lower educational attainment are more likely to drop out of a study. Lower study participation is seen in participants that have high levels of mental distress or heavy alcohol consumption, meaning participants with some of these characteristics tend to be underrepresented in health research. Therefore, addressing concerns about the generalizability of statistical evaluations made in studies may be

improved through a better understanding of item nonresponse behaviors. Identifying a genetic component of item nonresponse behavior could then help model studies around genotyped samples with data that is missing not at random.

There have been many studies focused on the genetic components of participation in scientific studies. The genetic underpinnings of item nonresponse behavior, however, remains mostly unknown. This study could provide insight about genetic variants associated with cognitive processes involved in item nonresponse. This study could also help establish a basis for further evaluating the impact of nonresponse bias on GWAS (see **question 3** for a definition of *GWAS*) of other disorders or traits.

3. **What is a GWAS?**

GWAS stands for genome-wide association study. GWAS is a widely used, established approach that allows scientists to test millions of genetic markers across the genome for associations with a specific trait systematically. Genetic differences among people can then be statistically analyzed to see, on average, which differences are associated with a certain outcome. For example, GWAS can be used to determine whether a particular DNA base (e.g., a G) at a specific location is associated with item nonresponse behavior.

4. **What are SNPs?**

SNPs stands for single-nucleotide polymorphisms – or places on the human genome where people normally have different base pairs (e.g., an A-T) or alleles. We look at any given SNP location on a chromosome – one chromosome is inherited for each parent. Therefore, we have inherited one allele at an SNP from each parent, meaning we would have two alleles total. Sometimes the two alleles are the same from each parent, sometimes they are different. These alleles can then be statistically evaluated by scientists for associations with certain characteristics, for example item nonresponse. SNPs evaluated through GWAS are normally only included if they are measurable with a high level of accuracy.

5. **Who conducted this study?**

The authors of this study are made up of scientists from universities, research institutes, and hospitals in the United States, Finland, Italy, Scotland, Sweden, and the Netherlands. The authors designed this study to help better understand genetic analyses in relation to nonresponse behaviors in survey research.

Findings

1. **Did you find the genetic basis of nonresponse behavior?**

We found a few places in the human genome that are highly associated with item nonresponse behavior. Each of these locations is associated with nonresponse, but their impact on biological processes is unknown and there is no evidence that they directly shape nonresponse in any way. While we identified these handful of locations, we did not discover a strong overall genetic component to item nonresponse behavior. There are no strong genetic predictors of item nonresponse behavior.

2. **What else did you find?**

In addition to the results highlighted above, we were also able to show preliminary evidence that nonresponse may contribute to overall biases in genetic signals from a GWAS. Future researchers may wish to investigate this finding further, especially as we continue to understand the way that different selective mechanisms like nonresponse may affect overall GWAS results.

3. **Do genes dictate the choices we make? Are people biologically predetermined to not respond to surveys?**

Like with many other complex social behaviors, there is evidence of an association with genetic variants. But this does not in any way mean that "genes are destiny." Rather, these small numbers of locations in the genome, in combination with social and environmental factors, are associated with an outcome like nonresponse behavior. Genes do not dictate the choices we make when responding to surveys, nor are individuals determined to respond to surveys in a particular way.

4. **What are some limitations of this study?**

This study included data from the UK Biobank, a health resource whose purpose is to improve the prevention, diagnosis, and treatment of a large variety of illnesses. Screening of cohort data for this study allowed for the final inclusion of about 360,000 people of European ancestry between the ages of 40 and 69. Both the ancestry and age biases in relation to the cohort demographics illustrate that this sample is not representative of the general population. This means that the study findings may not be applicable to people in other demographic categories (e.g., other ancestry groups).

Ethical & Social Implications of this Study

1. **What are the ethics of studying item nonresponse? What does studying item nonresponse have to do with participant consent?**

Participant consent is a crucial aspect of ethical research conduct. A participant may reflect their right to voluntarily not respond or engage in some aspects of a study in the form of a nonresponse to certain questionnaire items. This is especially true if participants actively select "prefer not to answer". Therefore, ethical consideration must be taken into account when attempting to evaluate how to study nonresponse without violating a participant's consent at the item level and the study level (informed consent).

There can be some ethical harm from ignoring the sources of missing data in research. The consideration of missingness is essential to identify the ways in which a study may not be representative of a population and could produce biased and ungeneralizable results.

2. **How was the participants' consent obtained for this study?**

Participants in the UK Biobank consented to having their deidentified data used in research "…that can support a diverse range of research intended to improve the prevention, diagnosis and treatment of illness, and the promotion of health throughout society" (https://www.ukbiobank.ac.uk/media/05ldg1ez/consent-form-uk-biobank.pdf).

There is no ability to link names or exact living locations of study participants to individual data in the UK Biobank. Further, because item nonresponse behavior is highly related to a number of health and health behavioral outcomes, the study is within the scope of appropriate uses of the data, which is included in our UK Biobank application 31063.

Nevertheless, because of the sensitive nature of this topic, we also sought permission for the specific scope of this paper (i.e., "to also study response rates and response characteristics (e.g., how often a response is left unanswered) and to examine whether there are any genetic factors that correlate with these response phenotypes"), which the UK Biobank granted under the same application.

Finally, we wished to also seek review of the project under our local Institutional Review Boards (IRBs). The details of those reviews can be found in our Ethical Approval Statement in the Methods.

3. **How did this study protect participants' right to privacy?**

In survey research, a participant's right to voluntarily not take part of a study is a critical component of ethical research, especially when respondents may choose not to answer questions about sensitive topics, like those about sexual orientation or mental health. Therefore, studying nonresponse takes careful consideration to ensure there are no breaches of this important contract between respondents and researchers.

In this paper, we evaluate group trends in overall item nonresponse. As we do this, we avoid exploring nonresponse behavior to single questions, and thus assure that no information can be used to associate results with any particular respondents, who themselves of course are already non identifiable due to the nature of data collection in the data we use. At one point in our study, in order to ensure that we are in fact avoiding analyses at the item level, we analyze a question about whether or not respondents remembered having a painful sunburn as a child, but we intentionally select this question as one that is socially less sensitive than other selections we may have picked.

Our analyses reflect a general behavioral tendency for someone to choose not to respond to survey items. Importantly, they are not reflective of nonresponse to any single, specific item. And to reiterate, no attempts were made to draw inferences about individual item-level responses in this study.

4. **Could future researchers explore nonresponse behavior to single questions?**

The ethical considerations in our study only apply to this specific study, and so it is possible that future research could head in this direction. However, such work would likely not be fruitful, given the low predictive power of overall nonresponse behavior at the individual-person, individual-item level. We very much encourage our colleagues to continue to remain vigilant to the ethical challenges surrounding genetics and nonresponse in all areas of this research domain.

5. **Should public officials, policy makers, insurers, or health care professionals use the results of this study to make decisions?**

No. Attempting to understand the causes of nonresponse has been a long-term concern for survey-based research. The factors that were generated for analysis in this study can be thought of as illustrating general behavior tendencies for those who choose not to respond to one or more survey items. These factors are designed solely for research use and are not a reflection of nonresponse to any specific, single item. The overall predictive power of our results are very small and are not directly transferable to other groups or datasets. Our results should not be used to meaningfully create policies or make decisions about particular individuals or groups of people.

SUPPLEMENTARY FIGURES

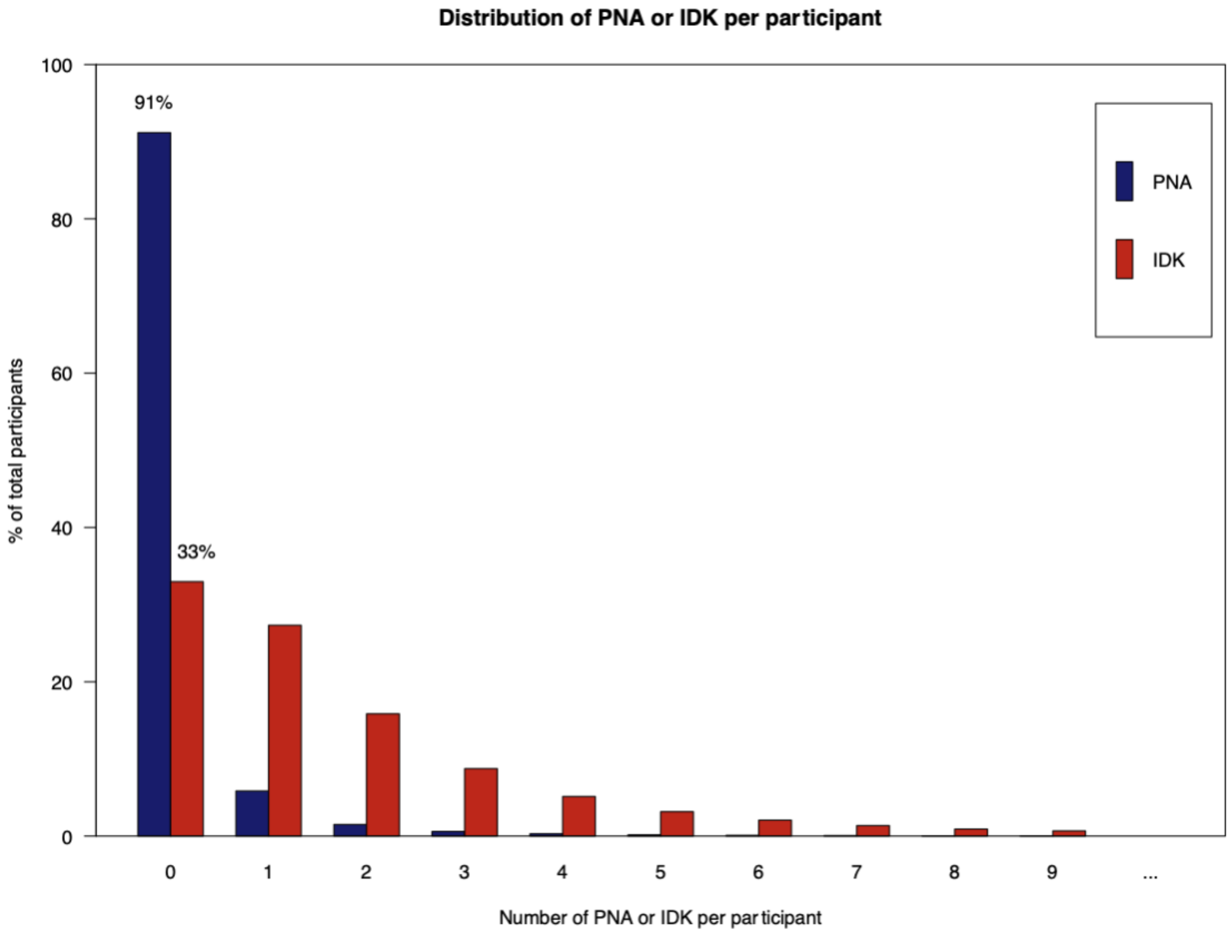

Supplementary Figure 1. Distribution of PNA and IDK per participant in the full UKB cohort

The plot highlights descriptives for different nonresponse behavior of participants in the PNA and IDK analyses. While 91% of the complete N=360,628 analytic sample reported no PNA throughout the UKB questionnaire, 33% reported no IDK throughout the UKB questionnaire. In other words, 9% of the sample preferred not to answer at least one question, whilst 67% of the same sample didn't know how to answer at least one question of the touchscreen questionnaire in UKB.

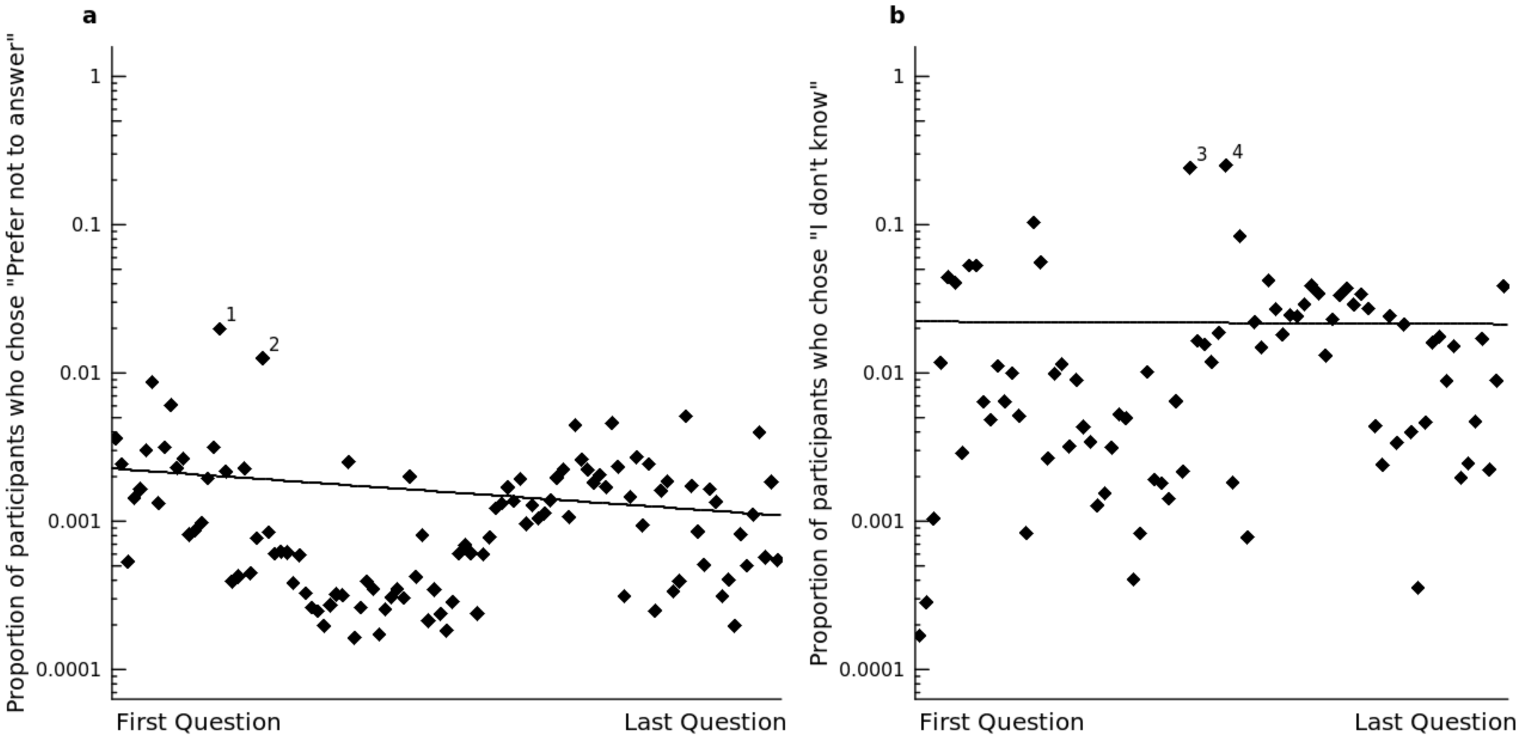

Supplementary Figure 2. Distribution of item nonresponse across 109 UK Biobank touchscreen questionnaire questions

Percentage of participants who chose the option "Prefer not to answer" (panel **a**) or "I don't know" (panel **b**). Questions have been ordered in the same order as they appeared in the questionnaire. The lines represent the fitted values from a negative binomial regression of the counts of PNA and IDK. Questions annotated are those with the highest number of nonresponses. **1**: "How often do you drive faster than the speed limit on the motorway?", **2**: "Does your partner or a close relative or friend complain about your snoring?", **3**: "Were you breastfed when you were a baby?", **4**: "Before the age of 15, how many times did you suffer sunburn that was painful for at least 2 days or caused blistering?"

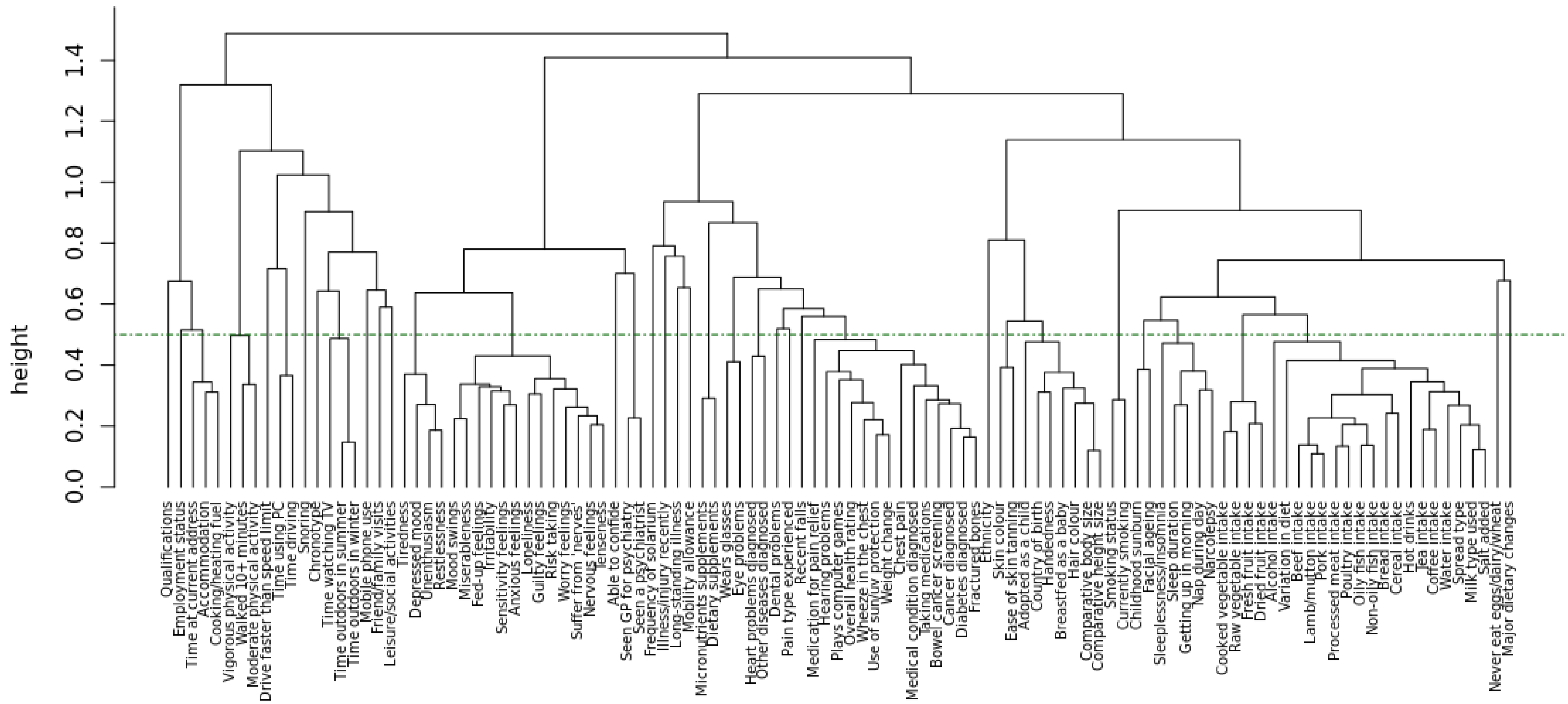

Supplementary Figure 3. Dendrogram of residuals from Factor Analysis with one factor for PNA

The dashed line at height 0.500 is the cut point that allowed us to reduce the number of questions to keep in the analysis.

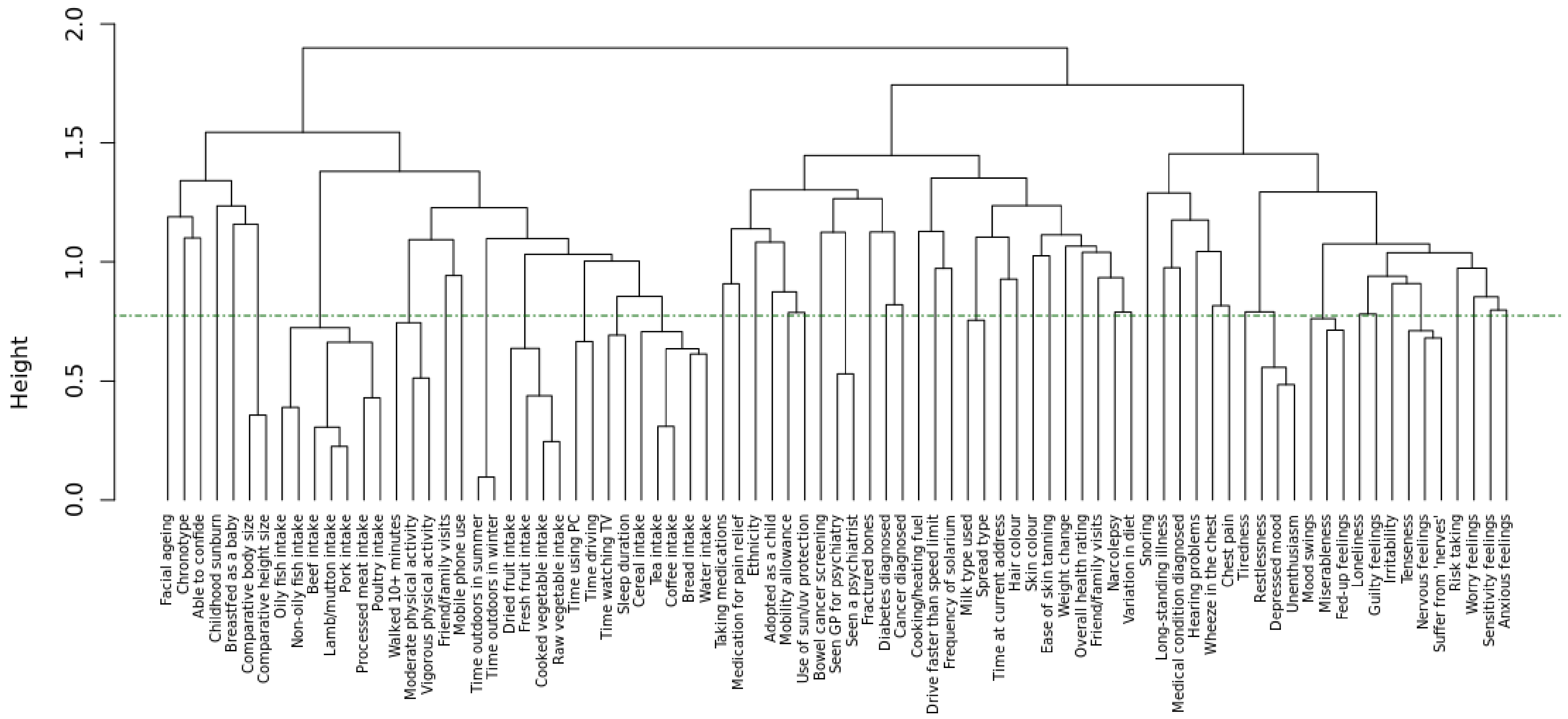

Supplementary Figure 4. Dendrogram of residuals from Factor Analysis with one factor in for IDK

The dashed line at height 0.775 is the cut point that allowed us to reduce the number of questions to keep in the analysis.

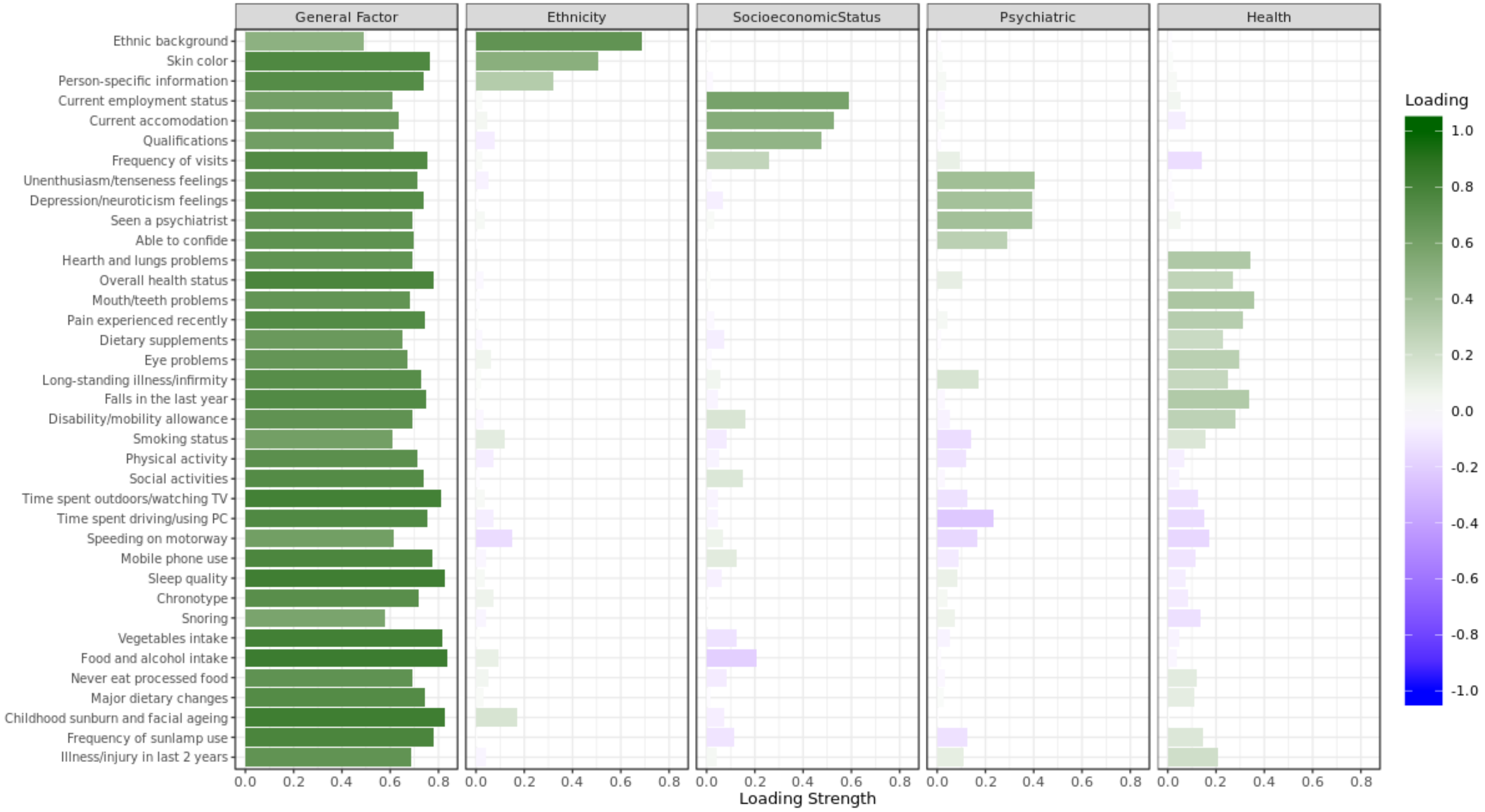

Supplementary Figure 5. Bar graph of factor loadings for questions in the PNA Exploratory Factor Analysis

The plot represents the loading strength of each question on the latent factors in the Exploratory Factor Analysis with a Bi-factor model in the "Prefer Not to Answer" analysis.

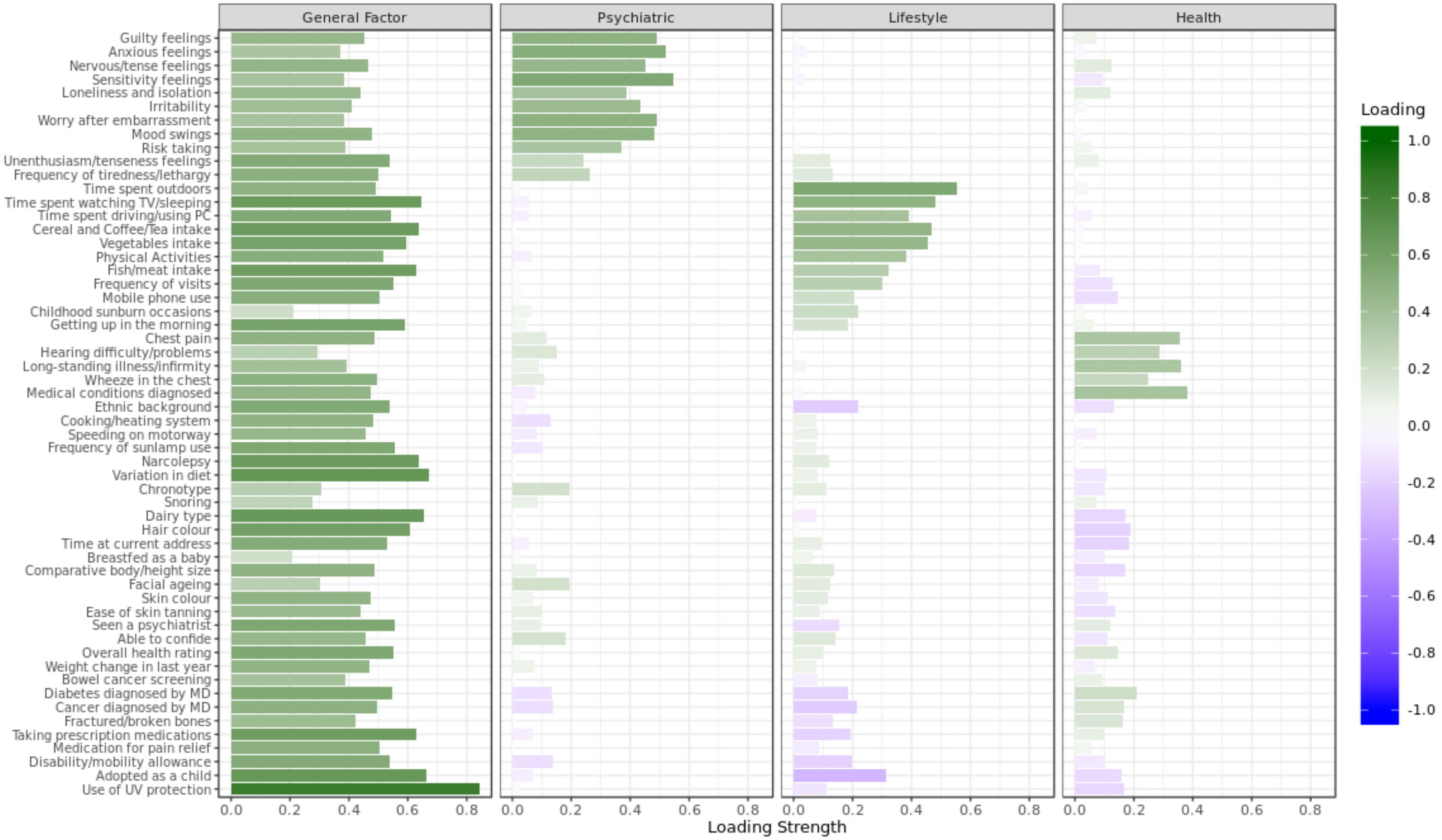

Supplementary Figure 6. Bar graph of factor loadings for questions in the IDK Exploratory Factor Analysis

The plot represents the loading strength of each question on the latent factors in the Exploratory Factor Analysis with a Bi-factor model in the “I Don’t Know” analysis.

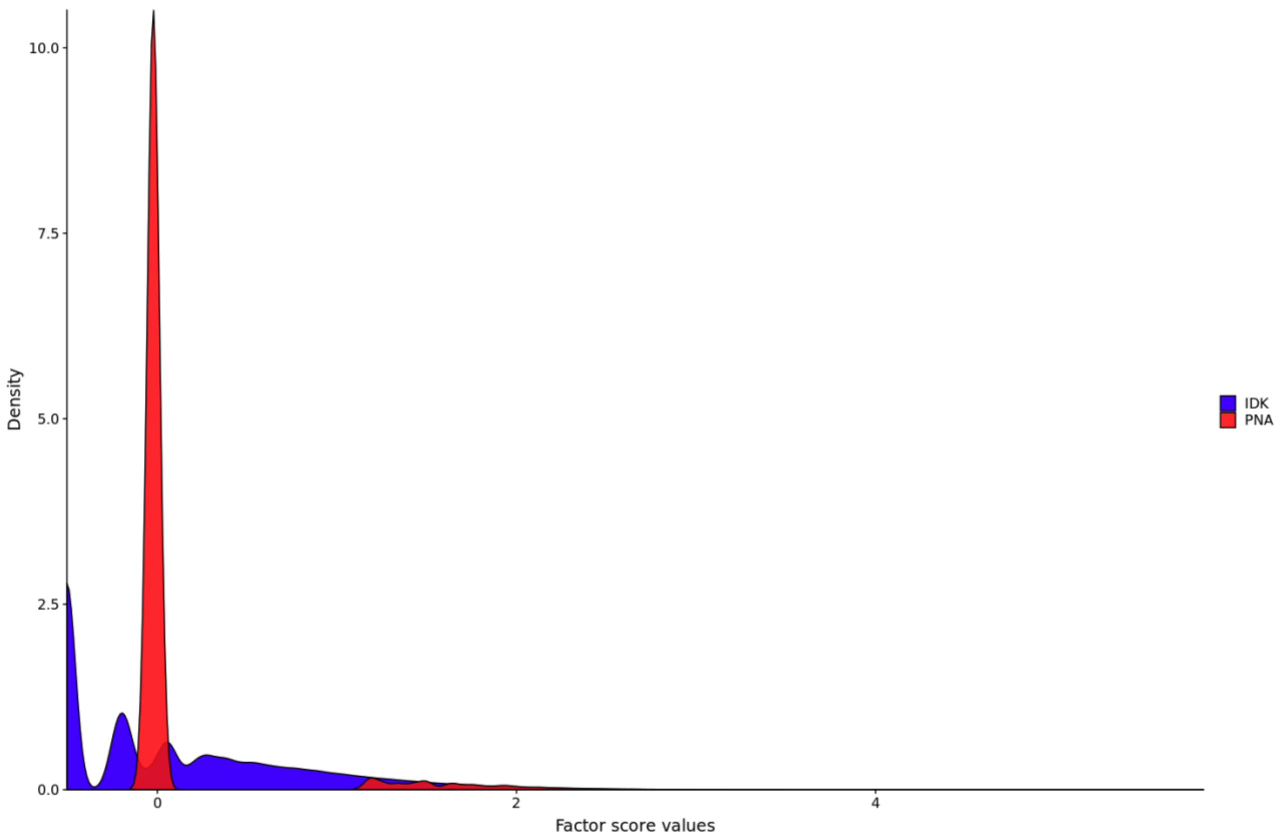

Supplementary Figure 7. Density plots of factor scores from the Confirmatory Factor Analysis for both PNA and IDK.

The plot shows the densities of the factor scores for the general latent factor from the Confirmatory Factor Analysis for both PNA and IDK in the touchscreen questionnaire in UKB. Factor scores for PNA mostly clustered around 0, while factor scores for IDK were more sparse, with less values clustered around 0.

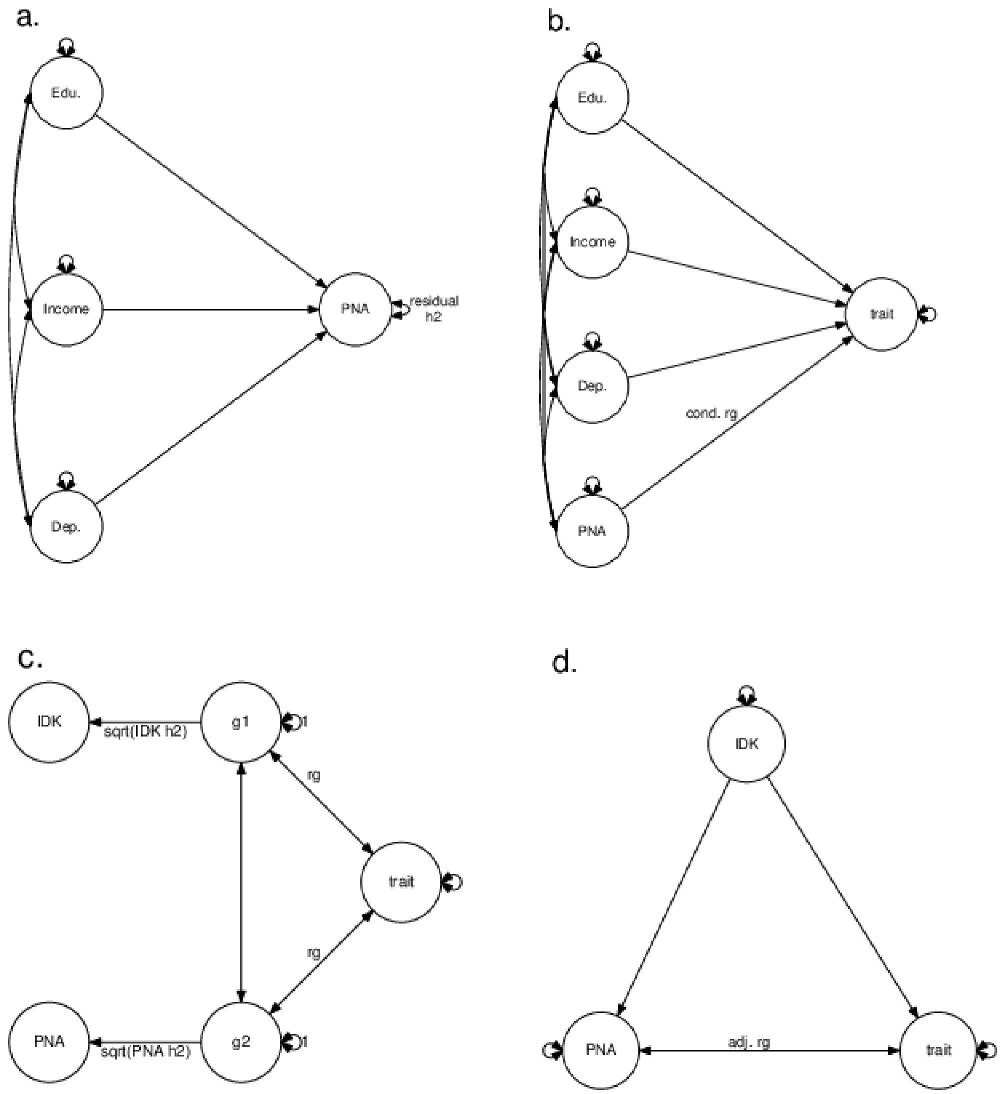

Supplementary Figure 8. Path diagrams for Genomic Structural Equation Models (SEM).

Example path diagrams for analyses of (a) residual h2 of nonresponse conditional on socioeconomic variables educational attainment (Edu.), household income, and regional social deprivation (Dep.); (b) conditional genetic correlation of nonresponse with other traits controlling for socioeconomic variables; (c) difference in genetic correlation of PNA and IDK with other traits; and (d) genetic correlation of nonresponse with other traits after controlling for the other nonresponse type (e.g. here IDK-adjusted PNA). The difference in genetic correlations with IDK and PNA was tested based on the misfit of the depicted model with r_g constrained to be equal (panel c). All other models tested for the indicated parameter.

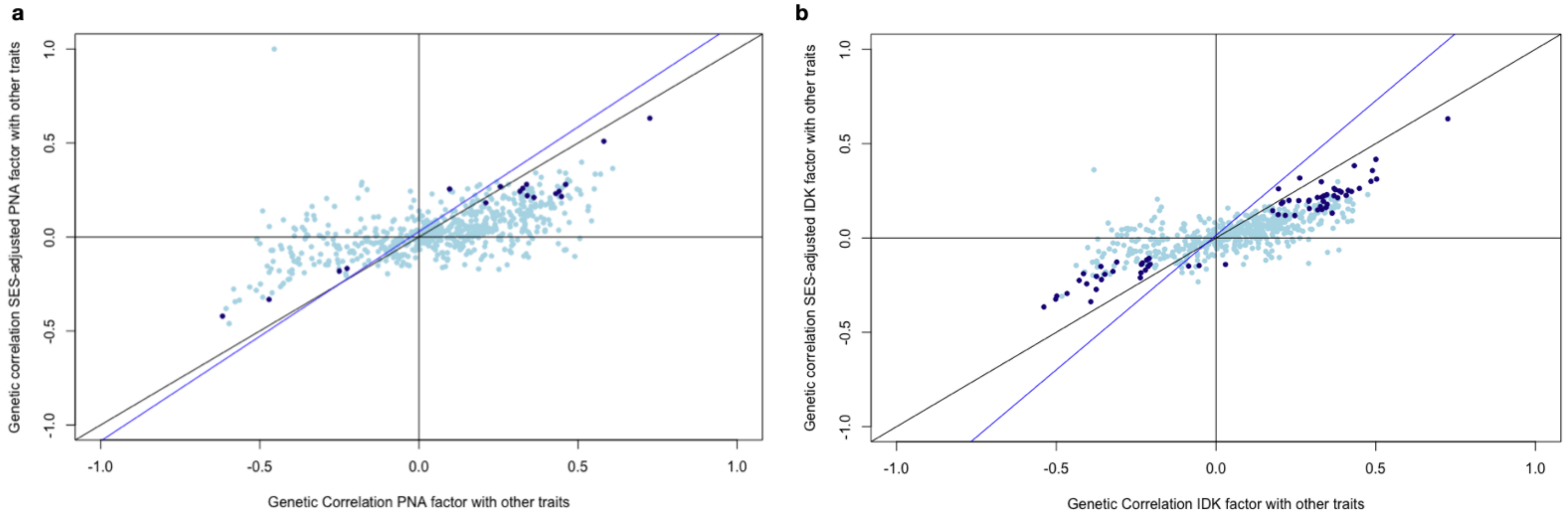

Supplementary Figure 9. Adjusted vs unadjusted genetic correlations between PNA/IDK factors and other heritable traits, adjusted for SES.

Comparison of the genetic correlation between PNA in panel **a** and IDK in panel **b** with other heritable traits, before and after (x and y axes, respectively) adjusting for Socioeconomic Status (SES), namely income and educational attainment, using Genomic SEM. Dots on the bisecting line suggest that the genetic correlation between PNA/IDK and other traits, after subtracting the effect of socioeconomic confounders, remains unchanged. Darker dots are those significant after a multiple hypothesis testing correction (α=0.05, N traits=654). The red lines are regression lines, interpolating the dots.

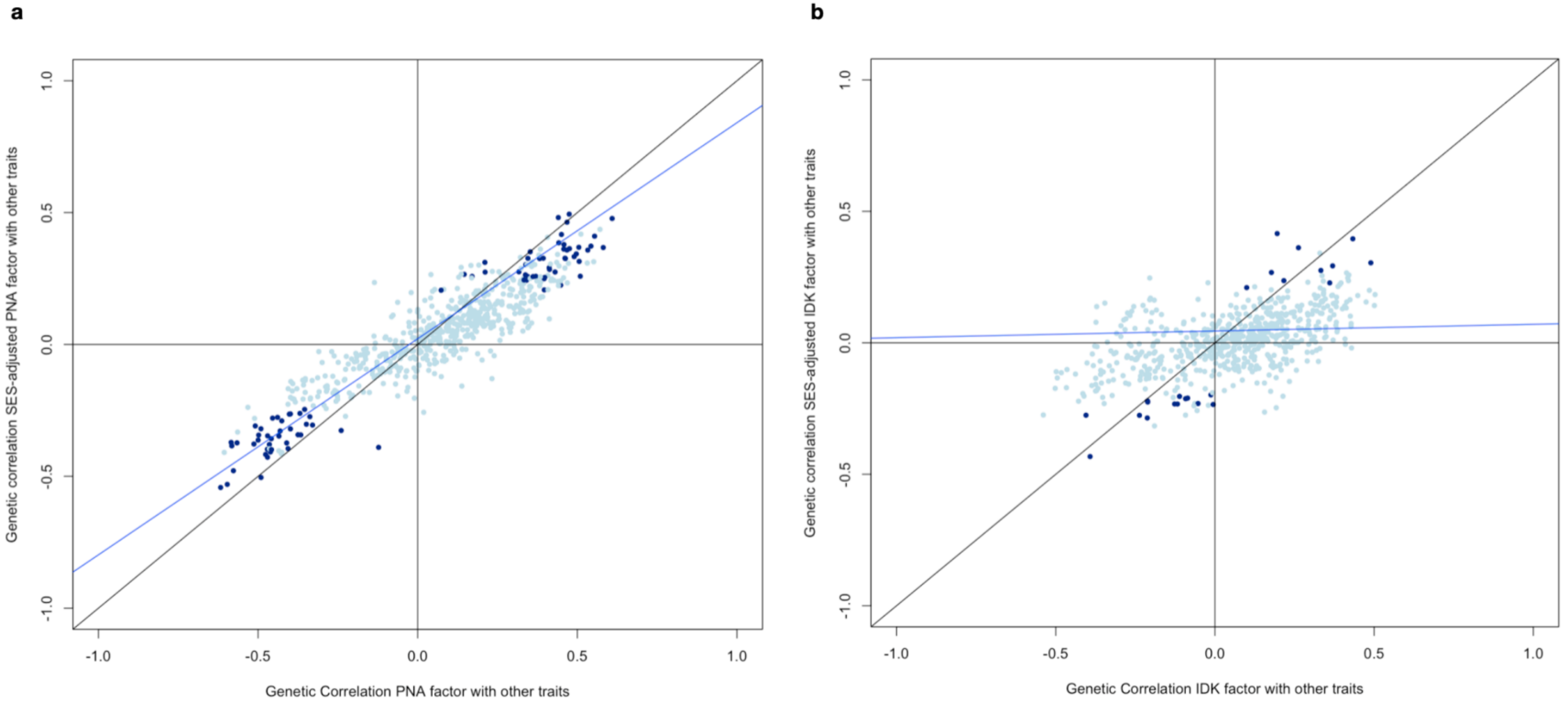

Supplementary Figure 10. Adjusted vs unadjusted genetic correlations between PNA/IDK factors and other heritable traits, adjusted for IDK/PNA, respectively

Comparison of the genetic correlation between PNA in panel **a** and IDK in panel **b** with other heritable traits, before and after (x and y axes, respectively) adjusting for PNA/IDK, respectively using Genomic SEM. By adjusting for PNA/IDK we remove the effect of PNA from IDK, and vice versa. Dots on the bisecting line suggest that the genetic correlation between PNA/IDK and other traits, after subtracting the effect of the other nonresponse trait, remains unchanged. Darker dots are those significant after a multiple hypothesis

testing correction (α =0.05, N traits=654). The blue lines are regression lines, interpolating the dots. Since the line in panel **a** is closer to the bisector line, this suggests that most of the genetic correlations between PNA and the other traits remained unchanged, both before and after subtracting the effect of IDK.

SUPPLEMENTARY REFERENCES

1. Little, R. J. A. & Rubin, D. B. *Statistical Analysis with Missing Data*. (John Wiley & Sons, Inc., 2002). doi:10.1002/9781119013563.
2. Aschard, H., Vilhjálmsson, B. J., Joshi, A. D., Price, A. L. & Kraft, P. Adjusting for heritable covariates can bias effect estimates in genome-wide association studies. *Am. J. Hum. Genet.* (2015) doi:10.1016/j.ajhg.2014.12.021.
3. Pirastu, N. *et al.* Genetic analyses identify widespread sex-differential participation bias. *Nat. Genet.* **53**, (2021).
4. Monsees, G. M., Tamimi, R. M. & Kraft, P. Genome-wide association scans for secondary traits using case-control samples. *Genet. Epidemiol.* **33**, 717–728 (2009).
5. Verhulst, B., Maes, H. H. & Neale, M. C. GW-SEM: A Statistical Package to Conduct Genome-Wide Structural Equation Modeling. *Behav. Genet.* **47**, 345–359 (2017).
6. Cai, S., Hartley, A., Mahmoud, O., Tilling, K. & Dudbridge, F. Adjusting for collider bias in genetic association studies using instrumental variable methods. *Genet. Epidemiol.* (2022) doi:10.1002/gepi.22455.
